# Analysis of Association between Adolescents’ Food Habits and Body Mass Change in a Population-Based Sample: Diet and Activity of Youth during COVID-19 (DAY-19) Study

**DOI:** 10.3390/ijerph191811772

**Published:** 2022-09-18

**Authors:** Aleksandra Kołota, Dominika Głąbska

**Affiliations:** Department of Dietetics, Institute of Human Nutrition Sciences, Warsaw University of Life Sciences (SGGW-WULS), 159c Nowoursynowska Street, 02-776 Warsaw, Poland

**Keywords:** adolescents, body mass, body mass change, dietary habits, physical activity, population-based study, national study, Adolescents’ Food Habits Checklist (AFHC), COVID-19, DAY-19 Study

## Abstract

The COVID-19 pandemic may have contributed to food habit changes, including some negative ones that may increase the risk of overweight and obesity. The aim of the study was to analyze the association between adolescents’ food habits, body mass change, as well as physical activity level in a population-based sample within the Diet and Activity of Youth during COVID-19 (DAY-19) Study. The DAY-19 Study was conducted in a cohort of 1333 students (aged 10–16 years) recruited in schools after stratified random quota sampling of primary schools (sampling counties within voivodeships and schools within counties) in June 2020. The food habits were assessed for the period of the COVID-19 pandemic and the period before the COVID-19 pandemic while using Adolescent Food Habits Checklist (AFHC). The body mass changes were assessed based on body weight and height for the period of the COVID-19 pandemic and the period before the COVID-19 pandemic while using Polish growth reference values. The physical activity changes were assessed based on the subjective assessment of adolescents. It was stated that for female adolescents declaring body mass gain during the COVID-19 pandemic the highest AFHC purchase scores (healthy purchase habits) (*p* < 0.0001) were accompanied by the lowest AFHC consumption score (unhealthy consumption habits) (*p* < 0.0001), as well as for female adolescents declaring physical activity decrease during the COVID-19 pandemic the highest AFHC purchase scores (healthy purchase habits) were observed (*p* = 0.0333). It was stated that for male adolescents declaring physical activity increased during the COVID-19 pandemic, the highest AFHC consumption scores (healthy consumption habits) were observed (*p* = 0.0003). In the case of a majority of participants, the general food habits were unchanged, which was observed mainly for food habits associated with food preparation. More food habits changes associated with the COVID-19 pandemic resulting in body mass changes were observed in females than in male adolescents. Body mass gain during the COVID-19 pandemic in adolescents may have resulted from unhealthy consumption habits, accompanied by decreased physical activity, in spite of the fact that this sub-group presented healthy purchase habits, which was observed especially for female adolescents.

## 1. Introduction

The novel pandemic situation, associated with SARS-CoV-2 virus transmission and resulting social isolation, caused numerous changes in the everyday lives of not only adults [1], but also children and adolescents [2]. The changes are associated with numerous aspects of everyday life, including physical, mental, and social health [3], resulting from loneliness, stress, anxiety, and depression [4]. Those changes resulted from the restrictions associated with social distance and home isolation, as well as from the social and economic consequences of the COVID-19 pandemic [5].

Among various consequences of the COVID-19 pandemic, there are changes in consumer behaviors aiming at reducing food-wasting, as well as saving and storing food properly [6]. At the same time, behaviors associated with grocery shopping during the COVID-19 pandemic were modified, because consumers intended to reduce the number of shopping trips, so they intended to purchase more during each store visit, in order to stock up with long shelf-life products, while trying to choose healthy options [7]. People declared a reduced number of shopping trips, accompanied by limited consumption of fresh food products, being replaced by processed ones (frozen, canned), but increased consumption of cakes and sweets [8]. The general food intake was also increased in a number of individuals, which resulted mainly from an increased intake of convenient foods, sweets, desserts, and salty snacks [9,10]. Additionally, in the Polish study, the increased consumption was declared by a number of respondents, including especially those with excessive body mass [11].

Considering numerous changes in consumer behaviors observed in the case of adults, it may be supposed, that similar changes may be stated for adolescents, as their nutritional behaviors during pandemics are influenced by similar determinants as for adults [12]. However, for adolescents, additional determinants are indicated or may have a stronger impact, including the influence of family, school, and media [13,14]. In spite of the fact that for adults there were more studies conducted, some studies published for children and adolescents confirm also increased consumption of sweets [15,16] and decreased consumption of fresh foods, including fruits and vegetables [15,17]. The previous own study conducted on a population of Polish primary school adolescents, within Diet and Activity of Youth during COVID-19 (DAY-19) Study, indicated some negative food habit changes during the COVID-19 pandemic, including decreased frequency of choosing a low-fat option, but at the same time, a number of food habits were changed to be more beneficial [18].

Taking into account the changes in food habits during the COVID-19 pandemic, which are observed not only for adults but also for adolescents, their consequences should be assessed. Such consequences may be associated mainly with body mass changes, as both increased consumption of sweets [19] and decreased consumption of fruits and vegetables is proven to result in the risk of overweight and obesity in children and adolescents [20]. At the same time, body mass increase may result from reduced physical activity level, which was common during the COVID-19 pandemic [21], but also less beneficial food habits are indicated to promote reduced physical activity [22]. Taking this into account, the aim of the study was to analyze the association between adolescents’ food habits, assessed while using the Adolescent Food Habits Checklist (AFHC), and body mass change, as well as physical activity level in a population-based sample of Polish secondary school students, recruited based on a stratified random quota sampling of schools.

## 2. Materials and Methods

### 2.1. Ethical Approval and Study Design

The study was conducted based on the approval of the Ethics Committee of the Institute of Human Nutrition Sciences of the Warsaw University of Life Sciences (WULS-SGGW) (18/2020), while all procedures were in agreement with the Declaration of Helsinki, and the participants, as well as their parents/legal guardians, provided informed consent to participate in the study.

The DAY-19 Study aimed to assess the nutritional behaviors and physical activity in a population-based sample of Polish primary school adolescents during the COVID-19 pandemic while comparing it with the nutritional behaviors and physical activity before the COVID-19 pandemic. The studied population was recruited based on a stratified random quota sampling of schools and students were invited to participate in the study within primary school population. The previous analysis conducted within the DAY-19 Study indicated that majority of nutritional behaviors in a Polish population of primary school adolescents were more beneficial [22], but the screen time was significantly higher during the COVID-19 pandemic than before [23].

### 2.2. DAY-19 Study Population

The sample size for the DAY-19 Study was calculated for the population of Polish adolescents aged 10–16 years (2,726,492, on the basis of the data of the Central Statistical Office (CSO) in Poland [24]), while the confidence level of 95% and margin of error of 5% were taken into account. Assuming a percentage of 50%, the required sample size was calculated as 384 respondents. Taking this into account, the sample of 1333 respondents was interpreted as sufficient.

As described in the previous studies [22,23], the DAY-19 Study population, aged 10–16 years, was recruited within schools participating in the study (all adolescents meeting the inclusion criteria were invited to participate), while the primary schools were recruited based on stratified random quota sampling within the national register of primary schools. The recruitment procedure was conducted in June 2020.

The stratified random quota sampling of primary schools was conducted in 2 stages: a sampling of counties within voivodeships, being administrative units of Poland (16 voivodeships in Poland; 10 counties selected for each voivodeship), and a sampling of schools within counties (160 counties selected in previous stage; 10 schools selected for each county), based on a commonly applied methodology [25].

Principal of each primary school sampled was invited to agree for his school to participate in the study, and if agreed, the students were invited to participate. All the students agreeing to participate received a link to an electronic version of the questionnaire (Computer-Assisted Web Interview (CAWI) method), while the questionnaire was developed in such a way to not allow to identify specific participating students, as well as it did not gather any personal and sensitive data, other than those necessary to verify inclusion and exclusion criteria.

The inclusion criteria for students were as follows:

− Being a student of the sampled primary school;
− Age of 10–16 years;
− Providing informed consent of students, as well as of parents/legal guardians to participate.

The exclusion criteria were as follows:

− Any missing data in the completed questionnaire;
− Any unreliable answers in the completed questionnaire.

The final number of schools participating in the study was 43, and the final number of adolescents participating in the study was 1333, differing from 6 to 254 per school (mean of 40 per school). The data gathering was conducted in June 2020.

### 2.3. Questionnaire

The questionnaire applied within the study consisted of 4 parts: confirming the inclusion criteria, assessing food habits, assessing body mass, and assessing physical activity. The questionnaire was based on simple questions, accompanied by instructions to provide as reliable answers as possible, as there are no wrong answers, they are not judged in any way, and they are needed for scientific research.

In order to confirm the inclusion criteria, students were asked about their school (to verify if it was sampled within sampling of schools), their age (to verify if the student is of the age of 10–16 years), and about the informed consent of student, as well as of parents/legal guardians (to verify if both student and parents/legal guardians agreed for participation).

In order to assess food habits, the validated AFHC by Johnson et al. [26] was applied. This questionnaire is commonly applied in various countries, to assess food habits of adolescents [27,28,29], including food habits changes during the COVID-19 pandemic [30,31]. It is based on self-reported data associated with food purchase, preparation, and consumption behaviors, assessed based on 23 simple questions [26]. The Polish version of the AFHC was developed on the basis of forward and backward translation, accompanied by a transcultural adaptation [30], as recommended by the World Health Organization (WHO) [32].

Within the DAY-19 Study, questions from AFHC were asked twice—for the period of COVID-19 pandemic (current situation) and period before COVID-19 pandemic. In order to allow easy recognition of the period before COVID-19 pandemic, adolescents were asked about the period before remote education, which should have been for them easy to distinguish from the period of remote education. Such an approach was possible, as in Poland, based on the decision of the Ministry of National Education from the beginning of COVID-19 (March 2020) the education in primary schools was changed into a remote system, or partially remote system (depending on the current COVID-19 situation) [33], and it was also applied in the other Polish studies in order to compare situation for the period of COVID-19 pandemic and for the period before COVID-19 pandemic [34]. Gathering the data describing pre-pandemic period during the COVID-19 pandemic may have been considered reliable, as for the studies conducted for children and adolescents for various behaviors, retrospective data are stated to be valid, including information about dietary behaviors [35] and physical activity behaviors [36]. Similarly, for adults, even longer recall periods are indicated as reliable for various behaviors, such as dietary behaviors [37] and physical activity behaviors [38].

The AFHC data were interpreted according to the recommendations by Johnson et al. [26], based on the food habits presented within the questionnaire, being defined as healthy, or unhealthy. Within each question, healthy behavior was scored as 1 point, and unhealthy behavior was scored as 0 points, so the total maximum scoring was 23 points, divided into maximum 6 points, 8 points, and 9 points, respectively, for purchase, preparation, and consumption habits [26]. The data were analyzed for continuous variables, and additionally after classification of respondents into categories. As commonly applied [27], the respondents were divided into quartiles, based on their scoring, while first quartile (the lowest scores) was defined as unhealthy habits, second and third combined (medium scores) were defined as neutral habits, and the fourth quartile (the highest scores) were defined as healthy habits. This approach was applied in order to evaluate the effect of the changes in specific food habits on general assessment of habits as healthy or unhealthy.

In order to assess body mass, simple questions about body weight and height were asked, and they were asked twice—for the period of COVID-19 pandemic (current situation) and period before COVID-19 pandemic, while for the period before COVID-19 pandemic, it was defined as the period before remote education. The respondents were asked about their body weight and height in the period directly before the COVID-19 pandemic, namely February 2020. Based on the obtained data, the body mass index (BMI) values were calculated based on Quetelet equation [39], separately for the period of COVID-19 pandemic and period before COVID-19 pandemic. Afterward, BMI values were interpreted based on the growth reference values developed for Polish children and adolescents, taking into account their gender and age [40], while using dedicated software [41]. Finally, the centile change was interpreted as body mass loss, body mass gain, or remaining stable body mass during the COVID-19 pandemic, while the change by no more than 2 BMI centiles was interpreted as stable body mass, a BMI increase of 3 centiles or more—as body mass gain, and a BMI decrease of 3 centiles or more—as body mass loss.

In order to assess physical activity, the simple question was asked if it decreased, increased, or remained stable during the COVID-19 pandemic, compared with the period before the COVID-19 pandemic, while the period before COVID-19 pandemic was defined as the period before remote education. The question about physical activity changes during the COVID-19 pandemic was based on the common assumption that children and adolescents are able to recognize such changes. The same approach is also applied by the other authors [42].

### 2.4. Statistical Analysis

The distributions were verified while using the Shapiro–Wilk test and due to nonparametric distributions, median values and interquartile range (IQR) are presented, while sub-groups were compared while using the Kruskal–Wallis one-way analysis of variance by ranks.

The analysis of covariance (ANCOVA) was conducted for the AFHC scores for place of residence, gender, age, body mass change, and physical activity change, as influencing factors, while due to nonparametric distribution of the results, the log-transformation was conducted. For the included factors the following stratification was applied: place of residence (city—village), gender (male—female), age (10–13 years—13–16 years), body mass change (body mass loss—stable body mass—body mass gain) and physical activity change (decreased—remained stable—increased).

The Statistica 13.3 (StatSoft Inc., Tulsa, OK, USA) and Jamovi 2.2.5 (The jamovi project, 2021, https://www.jamovi.org) (accessed on 3 September 2022) were used to conduct statistical analysis, and *p* ≤ 0.05 was defined as significant.

## 3. Results

The AFHC scores for the period before the COVID-19 pandemic and for the period of the COVID-19 pandemic, stratified by body mass changes during the COVID-19 pandemic, for the population of female adolescents from the DAY-19 Study are presented in Table 1. In the studied group, it was stated that AFHC purchase score and AFHC consumption score differed between subgroups stratified by body mass changes, while the differences were observed for the period before the COVID-19 pandemic only—for female adolescents declaring body mass gain during the COVID-19 pandemic the highest AFHC purchase scores (healthy purchase habits) (*p* < 0.0001) were accompanied by the lowest AFHC consumption score (unhealthy consumption habits) (*p* < 0.0001).

The AFHC scores for the period before the COVID-19 pandemic and for the period of COVID-19 pandemic, stratified by body mass changes during the COVID-19 pandemic, for the population of male adolescents from the DAY-19 Study are presented in Table 2. In the studied group, there were no statistically significant differences in AFHC scores between subgroups stratified by body mass changes (*p* > 0.05).

The AFHC scores for the period before the COVID-19 pandemic and for the period of COVID-19 pandemic, stratified by body mass changes during the COVID-19 pandemic, for the total population of adolescents from the DAY-19 Study are presented in Table 3. In the studied group, there were no statistically significant differences in AFHC scores between subgroups stratified by body mass changes (*p* > 0.05).

The AFHC scores for the period before the COVID-19 pandemic and for the period of COVID-19 pandemic, stratified by physical activity changes during the COVID-19 pandemic, for the population of female adolescents from the DAY-19 Study are presented in Table 4. In the studied group, it was stated that AFHC purchase score differed between subgroups stratified by physical activity changes, while the differences were observed for the period of the COVID-19 pandemic only—for female adolescents declaring physical activity decreased during the COVID-19 pandemic, the highest AFHC purchase scores (healthy purchase habits) were observed (*p* = 0.0333).

The AFHC scores for the period before the COVID-19 pandemic and for the period of COVID-19 pandemic, stratified by physical activity changes during the COVID-19 pandemic, for the population of male adolescents from the DAY-19 Study are presented in Table 5. In the studied group, it was stated that AFHC consumption score differed between subgroups stratified by physical activity changes, while the differences were observed for the period of COVID-19 pandemic only—for male adolescents declaring physical activity increased during the COVID-19 pandemic, the highest AFHC consumption scores (healthy consumption habits) were observed (*p* = 0.0003).

The AFHC scores for the period before the COVID-19 pandemic and for the period of COVID-19 pandemic, stratified by physical activity changes during the COVID-19 pandemic, for the total population of adolescents from the DAY-19 Study are presented in Table 6. In the studied group, there were no statistically significant differences in AFHC scores between subgroups stratified by physical activity changes (*p* > 0.05).

The ANCOVA for the AFHC total score for the period before the COVID-19 pandemic for the place of residence, gender, age, body mass change, and physical activity change in the total population of adolescents from the DAY-19 Study is presented in Supplementary Table S1. In the studied group, the statistical significance was observed only for the physical activity change as the influencing factor, while no covariance was revealed.

The ANCOVA for the AFHC purchase score for the period before the COVID-19 pandemic for the place of residence, gender, age, body mass change, and physical activity change in the total population of adolescents from the DAY-19 Study is presented in Supplementary Table S2. In the studied group, the statistical significance was observed only for the physical activity change as the influencing factor, while the covariance was revealed for the place of residence, age, body mass change, and physical activity change combined.

The ANCOVA for the AFHC preparation score for the period before the COVID-19 pandemic for the place of residence, gender, age, body mass change, and physical activity change in the total population of adolescents from the DAY-19 Study is presented in Supplementary Table S3. In the studied group, the statistical significance was observed only for the physical activity change as the influencing factor, while no covariance was revealed.

The ANCOVA for the AFHC consumption score for the period before the COVID-19 pandemic for the place of residence, gender, age, body mass change, and physical activity change in the total population of adolescents from the DAY-19 Study is presented in Supplementary Table S4. In the studied group the covariance was revealed for gender, body mass change, and physical activity change combined.

The ANCOVA for the AFHC total score for the period of the COVID-19 pandemic for the place of residence, gender, age, body mass change, and physical activity change in the total population of adolescents from the DAY-19 Study is presented in Supplementary Table S5. In the studied group, no statistical significance was observed, and no covariance was revealed.

The ANCOVA for the AFHC purchase score for the period of the COVID-19 pandemic for place of residence, gender, age, body mass change, and physical activity change in the total population of adolescents from the DAY-19 Study is presented in Supplementary Table S6. In the studied group, the covariance was revealed for gender and body mass change, combined.

The ANCOVA for the AFHC preparation score for the period of the COVID-19 pandemic for the place of residence, gender, age, body mass change, and physical activity change in the total population of adolescents from the DAY-19 Study is presented in Supplementary Table S7. In the studied group, no statistical significance was observed, and no covariance was revealed.

The ANCOVA for the AFHC consumption score for the period of the COVID-19 pandemic for the place of residence, gender, age, body mass change, and physical activity change in the total population of adolescents from the DAY-19 Study is presented in Supplementary Table S8. In the studied group, no statistical significance was observed, and no covariance was revealed.

The changes in AFHC scores during the COVID-19 pandemic, in categories of healthy, neutral, and unhealthy habits, for the population of female adolescents from the DAY-1 Study are presented in Table 7. In the studied group, it was observed that in the case of a majority of female participants, the general food habits were unchanged (67.8%), which was observed mainly for food habits associated with food preparation (68.8%).

The changes in AFHC scores during the COVID-19 pandemic, in categories of healthy, neutral, and unhealthy habits, for the population of male adolescents from the DAY-1 Study are presented in Table 8. In the studied group, it was observed that in the case of a majority of male participants, the general food habits were unchanged (78.6%), which was observed mainly for food habits associated with food preparation (78.6%).

The changes in AFHC scores during the COVID-19 pandemic, in categories of healthy, neutral, and unhealthy habits, for the total population of adolescents from the DAY-1 Study are presented in Table 9. In the studied group, it was observed that in the case of a majority of participants, the general food habits were unchanged (72.1%), which was observed mainly for food habits associated with food preparation (73.4%).

## 4. Discussion

The presented study aimed to analyze the association between adolescents’ food habits, assessed while using AFHC during the COVID-19 pandemic, and body mass change, as well as physical activity level in a population-based sample of Polish secondary school students. Within the presented study it was stated that more food habits changes associated with the COVID-19 pandemic resulting in body mass changes were observed in female than in male adolescents. Body mass gain during the COVID-19 pandemic in adolescents was associated mainly with improper consumption habits, even if proper purchase habits were observed. At the same time, decreased physical activity was observed during the COVID-19 pandemic.

The period of adolescence is associated with increasing independence of young people, including making their own decisions associated with lifestyle, as well as consuming products or dishes [43]. Apart from the individual determinants influencing choices of food products and food habits of adolescents, such as hunger, attractiveness, convenience, and availability of food products, the other important determinants are associated with family and school environment, as well specific factors, such as mood and own body image [44,45]. The food habits created during the period of adolescence, both healthy and unhealthy ones, may influence health, including both current health, and long-lasting health effects, even consequences observed in adulthood [29,46].

Food choices may be an important determinant of body mass, which may either promote excessive body mass (unhealthy food choices) or prevent it (healthy food choices), being crucial especially during the COVID-19 pandemic, as a problem of overweight and obesity in adolescents intensified in this period [12]. The majority of studies analyzing the determinants of excessive body mass during the COVID-19 pandemic assessed changes in body mass as influenced by changes in dietary behaviors and physical activity [47,48,49], but little is known about purchase behaviors of adolescents, especially during the COVID-19 pandemic [30,31]. Taking this into account, in the presented study, general food habits, including food purchase, preparation and consumption were assessed, based on the well-known AFHC [26], in order to analyze the associations between food habits, body mass changes and physical activity changes in Polish adolescents during the COVID-19 pandemic.

In the conducted study, there were no statistically significant associations between food habits and body mass changes in the total study group of adolescents, and in male adolescents, which is in agreement with the results observed by Taleb and Itani [29], as they also did not notice any association between AFHC results and excessive body mass in Lebanese adolescents. However, in our own study, such associations were observed for the sub-population of female respondents, indicating that they may be more prone than male adolescents to some dietary behaviors influencing their body mass, as female adolescents declaring body mass gain during the COVID-19 pandemic were characterized by the lowest AFHC consumption score, indicating unhealthy habits. It may be explained by a number of factors that may influence obesogenic behaviors in female adolescents and women, including gender norms, failure to recognize the role of healthy foods, abundance, and promotion of cheap but unhealthy foods, food safety concerns, taste preferences, and social desirability of specific foods [50].

At the same time, in the presented own study, it was indicated that female adolescents declaring body mass gain during the COVID-19 pandemic were characterized not only by unhealthy food consumption habits but also by the highest AFHC purchase scores, indicating healthy purchase habits. It means that even healthy food purchase habits may have not improved unhealthy food consumption habits and that healthy food purchase habits accompanied by unhealthy food consumption habits still may lead to increased body mass. It may result from the fact that some unhealthy food consumption habits observed before the pandemic, were maintained during the pandemic, as was indicated by other authors [12,15,16]. As there are no studies published so far describing food purchase habits of adolescents during the COVID-19 pandemic, the results of our own study must be compared with the results of the studies conducted in the adult populations, which indicate general changes in food purchase behaviors, including changes in purchase patterns, intentions, and approach, that was observed in various countries [51], including Poland [52].

The changes in food purchase habits during the COVID-19 pandemic may result from the perceived crisis, associated with a worsened financial situation [51], especially important for consumers, if they lost their jobs, or if their incomes decreased [53]. The lockdown also may have caused changing approach to food purchase, which resulted from both financial factors, and the need to reduce social contacts, as consumers were reducing the number of shopping trips while increasing the number of food products purchased, but with the intention to reduce food wasting [54]. It may be supposed that such an approach associated with conscious consumerism may have been also presented by the parents of studied adolescents and may have been associated with more health-promoting purchase habits within the household, and being focused on food purchases, common during the COVID-19 pandemic [55], but it did not overcome the negative food consumption habits.

Before the COVID-19 pandemic, the important determinants of food habits of adolescents may have been associated with school, including the influence of peers [56], and food products available in schools (school lunch, shops, and vending machines) [57,58]. It may have been also associated with the influence of fast-food restaurants located near schools, which caused reduced fruit and vegetable consumption [59,60]. However, during the COVID-19 pandemic, during lockdowns and remote learning, the influence of school should have been reduced, which may have positively changed food habits.

At the same time, it may be supposed that lockdowns and remote learning should have reduced the physical activity of adolescents [21], which was observed in the studies conducted in Germany [61], Italy [62], France [63], Greece [64], and Poland [65]. Physical activity may be associated with food habits, as was observed in the study by Hermans et al. [45], as healthy food habits were becoming important for those adolescents who observed unfavorable changes in their physical fitness and wanted to overcome them. The associations between physical activity and food habits were observed also in the studied group of adolescents during the COVID-19 pandemic, but they were gender-dependent. In female adolescents, the associations were similar to those in the study by Hermans et al. [45], namely for female adolescents declaring physical activity decreased during the COVID-19 pandemic, the highest AFHC purchase scores (healthy purchase habits) were observed, which may be associated with healthy food habits becoming important for those adolescents who observe unfavorable changes in their physical fitness.

On the contrary, for male adolescents declaring physical activity increase during the COVID-19 pandemic the highest AFHC consumption scores (healthy consumption habits) were observed. There are two major differences while compared female and male adolescents in the presented own study. First, one result from the area of food habits associated with physical activity—for female adolescents the purchase habits were affected, while for male adolescents the consumption habits were affected. The other difference is associated with type of influence—while for girls, it may be supposed that decreased physical fitness motivates them to improve their diet, as in the study by Hermans et al. [45], for boys, it may be supposed that higher level of physical activity motivates them to have a better diet. The mechanism observed in the presented study for male adolescents may be also confirmed by the studies of the other authors, as it is stated that athletic adolescents present healthier food habits than non-athletic ones [66,67]. Such difference may be explained by the different roles of physical activity for boys and girls—as boys are in general more physically active than girls [68] and overestimate their level more than girls [69], it may be supposed that physical activity may be for them more important and they may present some actions to increase their physical fitness, while for girls only decreasing fitness may motivate them to counteract it.

Taking into account the aim of the study, its implications may be indicated. As the study investigated the association between adolescents’ food habits and body mass change, as well as physical activity level, in a population-based sample of Polish secondary school students, it must be emphasized that the major implications are associated with the novel knowledge in the described area, which was not studied so far. Moreover, also the practical implications should be indicated, as associated with the possibility to solve real-life problems resulting from body mass changes observed in adolescents during the COVID-19 pandemic. Based on the unhealthy consumption habits observed in adolescents gaining weight, accompanied by decreased physical activity, in spite of the healthy purchase habits, the areas of necessary nutritional education may be indicated.

In spite of the fact that the study presented novel observations associated with the body mass and food habits changes during the COVID-19 pandemic, the limitations of the study must be listed. The study was conducted in a population-based sample of 1333 individuals, but while stratified by the studied variables, the size of sub-groups was reduced, so conducting a study in a larger sample would allow for obtaining more representative results. Moreover, the studied individuals were stratified by some factors (gender, age, place of residence, body mass changes, physical activity changes), but there are also other factors that may have influenced the observed results, but were not taken into account, e.g., socioeconomic variables.

## 5. Conclusions

In the studied group, more food habit changes associated with COVID-19 pandemic resulting in body mass changes were observed in female than in male adolescents. Body mass gain during the COVID-19 pandemic in adolescents may have resulted from unhealthy consumption habits, accompanied by decreased physical activity, in spite of the fact that this sub-group presented healthy purchase habits, which in the studied group was observed especially for female adolescents.

## Figures and Tables

**Table 1:** The Adolescents’ Food Habits Checklist (AFHC) scores for the period before the COVID-19 pandemic and for the period of COVID-19 pandemic, stratified by body mass changes during the COVID-19 pandemic, for the population of female adolescents from the Diet and Activity of Youth during COVID-19 (DAY-19) Study (*n* = 710).

AFHC Scores	Body Mass Loss (*n* = 157)	Stable Body Mass (*n* = 285)	Body Mass Gain (*n* = 268)	*p*
AFHC for the period before COVID-19 pandemic				
AFHC total score	14.24 (10.45–17.77)	14.00 (10.00–17.77) *	14.64 (9.41–17.77) *	0.3340
AFHC purchase score	3.00 (2.00–4.50) *	3.00 (2.00–4.00) *	6.00 (2.00–4.50) *	<0.0001
AFHC preparation score	5.00 (3.43–7.00) *	5.00 (3.43–6.67) *	5.00 (3.00–6.67) *	0.5750
AFHC consumption score	6.00 (4.50–7.00) *	6.00 (4.00–7.87) *	3.00 (4.00–7.00) *	<0.0001
AFHC for the period of COVID-19 pandemic				
AFHC total score	14.95 (11.50–18.82)	15.33 (10.95–18.62)	15.33 (11.00–18.62) *	0.5033
AFHC purchase score	3.60 (2.40–4.80)	3.00 (2.40–4.50)	3.60 (2.00–4.80) *	0.3505
AFHC preparation score	5.00 (4.00–7.00)	5.00 (4.00–6.86)	5.33 (4.00–6.86) *	0.6007
AFHC consumption score	6.75 (5.00–8.00)	6.75 (4.50–7.87)	6.21 (5.00–7.87) *	0.7354

* nonparametric distribution.

**Table 2:** The Adolescents’ Food Habits Checklist (AFHC) scores for the period before the COVID-19 pandemic and for the period of COVID-19 pandemic, stratified by body mass changes during the COVID-19 pandemic, for the population of male adolescents from the Diet and Activity of Youth during COVID-19 (DAY-19) Study (*n* = 623).

AFHC Scores	Body Mass Loss (*n* = 86)	Stable Body Mass (*n* = 267)	Body Mass Gain (*n* = 270)	*p*
AFHC for the period before COVID-19 pandemic				
AFHC total score	14.00 (10.00–16.89) *	13.00 (9.41–17.52) *	13.45 (9.52–17.19) *	0.5928
AFHC purchase score	3.00 (2.00–4.37) *	3.00 (2.00–4.50) *	3.00 (2.00–4.00) *	0.9596
AFHC preparation score	4.90 (3.00–6.00) *	4.57 (3.00–6.40) *	4.57 (3.00–6.00) *	0.8520
AFHC consumption score	6.00 (4.00–7.54) *	5.00 (4.00–7.79) *	6.00 (4.00–7.00) *	0.0764
AFHC for the period of COVID-19 pandemic				
AFHC total score	15.33 (10.05–16.99) *	14.24 (10.45–18.62) *	14.64 (11.00–18.16) *	0.4415
AFHC purchase score	3.00 (2.00–4.00) *	3.00 (2.00–4.80) *	3.00 (2.40–4.80) *	0.8740
AFHC preparation score	5.00 (3.05–6.50) *	5.00 (3.43–6.86) *	5.00 (3.43–6.86) *	0.8212
AFHC consumption score	6.75 (4.00–7.00) *	6.00 (5.00–7.87) *	6.00 (4.50–7.87) *	0.2633

* nonparametric distribution.

**Table 3:** The Adolescents’ Food Habits Checklist (AFHC) scores for the period before the COVID-19 pandemic and for the period of COVID-19 pandemic, stratified by body mass changes during the COVID-19 pandemic, for the total population of adolescents from the Diet and Activity of Youth during COVID-19 (DAY-19) Study (*n* = 1333).

AFHC Scores	Body Mass Loss (*n* = 243)	Stable Body Mass (*n* = 552)	Body Mass Gain (*n* = 538)	*p*
AFHC for the period before COVID-19 pandemic				
AFHC total score	14.00 (10.23–17.52)	13.80 (9.86–17.52) *	14.00 (9.41–17.52) *	0.7007
AFHC purchase score	3.00 (2.00–4.50) *	3.00 (2.00–4.00) *	3.00 (2.00–4.50) *	0.8097
AFHC preparation score	5.00 (3.20–6.86) *	5.00 (3.43–6.47) *	5.00 (3.00–6.40) *	0.4451
AFHC consumption score	6.00 (4.00–7.00) *	6.00 (4.00–7.87) *	6.00 (4.00–7.00) *	0.2452
AFHC for the period of COVID-19 pandemic				
AFHC total score	15.00 (10.92–18.62) *	14.92 (10.89–18.62) *	15.00 (11.00–18.56) *	0.7602
AFHC purchase score	3.60 (2.00–4.80) *	3.00 (2.00–4.80) *	3.00 (2.00–4.80) *	0.6651
AFHC preparation score	5.00 (4.00–7.00) *	5.00 (3.43–6.86) *	5.33 (3.43–6.86) *	0.6378
AFHC consumption score	6.75 (5.00–7.87) *	6.00 (4.50–7.87) *	6.00 (5.00–7.87) *	0.5988

* nonparametric distribution.

**Table 4:** The Adolescents’ Food Habits Checklist (AFHC) scores for the period before the COVID-19 pandemic and for the period of COVID-19 pandemic, stratified by physical activity changes during the COVID-19 pandemic, for the population of female adolescents from the Diet and Activity of Youth during COVID-19 (DAY-19) Study (*n* = 710).

AFHC Scores	Decreased (*n* = 217)	Remain Stable (*n* = 225)	Increased (*n* = 268)	*p*
AFHC for the period before COVID-19 pandemic				
AFHC total score	14.64 (10.45–18.16) *	14.24 (9.68–17.25) *	14.00 (9.81–17.25) *	0.4455
AFHC purchase score	3.60 (2.00–4.50) *	3.00 (2.00–4.50) *	3.00 (2.00–4.00) *	0.1043
AFHC preparation score	5.00 (3.43–6.86) *	5.00 (3.43–6.67) *	5.00 (3.15–6.67) *	0.2002
AFHC consumption score	6.00 (5.00–7.87) *	6.00 (4.00–7.00) *	6.00 (4.00–7.00) *	0.7300
AFHC for the period of COVID-19 pandemic				
AFHC total score	15.68 (11.00–18.94) *	15.68 (10.95–18.40)	15.33 (11.00–18.28) *	0.1552
AFHC purchase score	3.60 (2.40–4.80) *	3.60 (2.00–4.80)	3.60 (2.30–4.50) *	0.0333
AFHC preparation score	5.71 (4.00–7.00) *	5.71 (4.00–6.86)	5.33 (4.00–7.00) *	0.0747
AFHC consumption score	6.00 (5.00–8.00) *	6.75 (5.00–7.87)	6.21 (4.87–7.87) *	0.7124

* nonparametric distribution.

**Table 5:** The Adolescents’ Food Habits Checklist (AFHC) scores for the period before the COVID-19 pandemic and for the period of COVID-19 pandemic, stratified by physical activity changes during the COVID-19 pandemic, for the population of male adolescents from the Diet and Activity of Youth during COVID-19 (DAY-19) Study (*n* = 623).

AFHC Scores	Decreased (*n* = 259)	Remain Stable (*n* = 165)	Increased (*n* = 199)	*p*
AFHC for the period before COVID-19 pandemic				
AFHC total score	13.00 (10.45–17.25) *	13.00 (8.76–17.77) *	14.64 (9.41–16.73) *	0.1866
AFHC purchase score	3.00 (2.00–4.00) *	3.00 (1.50–4.00) *	3.00 (2.00–4.50) *	0.2476
AFHC preparation score	4.80 (3.43–6.00) *	4.00 (3.00–6.67) *	5.00 (3.00–6.00) *	0.1082
AFHC consumption score	6.00 (4.25–7.87) *	5.00 (4.00–7.00) *	6.00 (3.00–7.00) *	0.5040
AFHC for the period of COVID-19 pandemic				
AFHC total score	14.00 (11.50–18.62) *	15.33 (9.36–18.40) *	15.33 (10.67–18.08) *	0.1120
AFHC purchase score	3.00 (2.20–4.80) *	3.00 (2.00–4.50) *	3.00 (2.20–4.80) *	0.4330
AFHC preparation score	5.00 (3.43–6.86) *	5.00 (3.00–6.86) *	5.71 (3.43–6.86) *	0.2249
AFHC consumption score	6.00 (5.00–7.87) *	6.00 (4.00–7.87) *	6.75 (4.50–7.87) *	0.0003

* nonparametric distribution.

**Table 6:** The Adolescents’ Food Habits Checklist (AFHC) scores for the period before the COVID-19 pandemic and for the period of COVID-19 pandemic, stratified by physical activity changes during the COVID-19 pandemic, for the total population of adolescents from the Diet and Activity of Youth during COVID-19 (DAY-19) Study (*n* = 1333).

AFHC Scores	Decreased (*n* = 476)	Remain Stable (*n* = 390)	Increased (*n* = 467)	*p*
AFHC for the period before COVID-19 pandemic				
AFHC total score	13.59 (10.45–17.80) *	14.00 (9.20–17.52) *	14.24 (9.41–17.00) *	0.6723
AFHC purchase score	3.00 (2.00–4.50) *	3.00 (2.00–4.50) *	3.12 (2.00–4.00) *	0.5346
AFHC preparation score	5.00 (3.43–6.47) *	5.00 (3.00–6.67) *	5.00 (3.00–6.67) *	0.7357
AFHC consumption score	6.00 (4.50–7.86) *	6.00 (4.00–7.00) *	6.00 (3.69–7.00) *	0.7262
AFHC for the period of COVID-19 pandemic				
AFHC total score	14.88 (11.50–18.82) *	15.51 (10.45–18.40) *	14.95 (10.95–18.16) *	0.1638
AFHC purchase score	3.00 (2.40–4.80) *	3.00 (2.00–4.80) *	3.00 (2.20–4.65) *	0.7119
AFHC preparation score	5.00 (3.43–6.86) *	5.33 (3.43–6.86) *	5.00 (3.43–6.86) *	0.4248
AFHC consumption score	6.00 (5.00–8.00) *	6.75 (4.50–7.86) *	6.75 (4.50–7.86) *	0.1262

* nonparametric distribution.

**Table 7:** The changes in Adolescents’ Food Habits Checklist (AFHC) scores during the COVID-19 pandemic, in categories of healthy, neutral, and unhealthy habits, for the population of female adolescents from the Diet and Activity of Youth during COVID-19 (DAY-19) Study (*n* = 710).

Habits		Measure of Habits				
	Before Pandemic (Baseline Habits)	During Pandemic (Final Habits)	AFHC Total Score	AFHC Purchase Score	AFHC Preparation Score	AFHC Consumption Score
Unchanged habits	Unhealthy	Unhealthy	109 (15.3%)	100 (14.1%)	116 (16.3%)	105 (14.8%)
	Neutral	Neutral	250 (35.2%)	228 (32.1%)	253 (35.6%)	237 (33.4%)
	Healthy	Healthy	123 (17.3%)	112 (15.8%)	120 (16.9%)	107 (15.1%)
	Total unchanged		482 (67.8%)	440 (62.0%)	489 (68.8%)	449 (63.3%)
Improved habits	Unhealthy	Neutral	53 (7.5%)	66 (9.3%)	49 (6.9%)	54 (7.6%)
	Neutral	Healthy	39 (5.5%)	54 (7.6%)	45 (6.3%)	52 (7.3%)
	Unhealthy	Healthy	15 (2.1%)	11 (1.5%)	12 (1.7%)	18 (2.5%)
	Total improved		107 (15.1%)	131 (18.4%)	106 (14.9%)	124 (17.4%)
Worsened habits	Neutral	Unhealthy	67 (9.4%)	74 (10.4%)	58 (8.2%)	67 (9.4%)
	Healthy	Neutral	53 (7.5%)	62 (8.7%)	54 (7.6%)	65 (9.2%)
	Healthy	Unhealthy	1 (0.1%)	3 (0.4%)	3 (0.4%)	5 (0.7%)
	Total worsened		121 (17.0%)	139 (19.5%)	115 (16.2%)	137 (19.3%)

**Table 8:** The changes in Adolescents’ Food Habits Checklist (AFHC) scores during the COVID-19 pandemic, in categories of healthy, neutral, and unhealthy habits, for the population of male adolescents from the Diet and Activity of Youth during COVID-19 (DAY-19) Study (*n* = 623).

Habits		Measure of Habits				
	Before Pandemic (Baseline Habits)	During Pandemic (Final Habits)	AFHC Total Score	AFHC Purchase Score	AFHC Preparation Score	AFHC Consumption Score
Unchanged habits	Unhealthy	Unhealthy	119 (19.1%)	109 (17.5%)	121 (19.4%)	81 (13.0%)
	Neutral	Neutral	248 (39.8%)	236 (37.9%)	248 (39.8%)	171 (27.4%)
	Healthy	Healthy	123 (19.7%)	118 (18.9%)	121 (19.4%)	79 (12.7%)
	Total unchanged		490 (78.6%)	463 (74.3%)	490 (78.6%)	331 (53.1%)
Improved habits	Unhealthy	Neutral	31 (5.0%)	40 (6.4%)	29 (4.6%)	66 (10.6%)
	Neutral	Healthy	27 (4.3%)	31 (5.0%)	29 (4.6%)	68 (10.9%)
	Unhealthy	Healthy	6 (1.0%)	7 (1.1%)	6 (1.0%)	9 (1.4%)
	Total improved		64 (10.3%)	78 (12.5%)	64 (10.2%)	143 (22.9%)
Worsened habits	Neutral	Unhealthy	36 (5.8%)	44 (7.1%)	34 (5.45%)	72 (11.6%)
	Healthy	Neutral	32 (5.1%)	35 (5.6%)	34 (5.45%)	74 (11.8%)
	Healthy	Unhealthy	1 (0.2%)	3 (0.5%)	1 (0.2%)	3 (0.5%)
	Total worsened		69 (11.1%)	82 (13.2%)	69 (11.1%)	149 (23.9%)

**Table 9:** The changes in Adolescents’ Food Habits Checklist (AFHC) scores during the COVID-19 pandemic, in categories of healthy, neutral, and unhealthy habits, for the total population of adolescents from the Diet and Activity of Youth during COVID-19 (DAY-19) Study (*n* = 1333).

Habits		Measure of Habits				
	Before Pandemic (Baseline Habits)	During Pandemic (Final Habits)	AFHC Total Score	AFHC Purchase Score	AFHC Preparation Score	AFHC Consumption Score
Unchanged habits	Unhealthy	Unhealthy	226 (16.9%)	136 (10.2%)	238 (17.8%)	219 (16.4%)
	Neutral	Neutral	494 (37.0%)	355 (26.6%)	501 (37.6%)	467 (35.0%)
	Healthy	Healthy	243 (18.2%)	150 (11.2%)	240 (18.0%)	214 (16.0%)
	Total unchanged		963 (72.1%)	641 (48.0%)	978 (73.4%)	900 (67.4%)
Improved habits	Unhealthy	Neutral	85 (6.4%)	162 (12.1%)	77 (5.8%)	89 (6.7%)
	Neutral	Healthy	68 (5.1%)	148 (11.1%)	75 (5.6%)	94 (7.0%)
	Unhealthy	Healthy	22 (1.6%)	35 (2.6%)	18 (1.3%)	25 (1.9%)
	Total improved		175 (13.1%)	345 (25.8%)	170 (12.7%)	208 (15.6%)
Worsened habits	Neutral	Unhealthy	105 (7.9%)	164 (12.3%)	91 (6.8%)	106 (7.9%)
	Healthy	Neutral	88 (6.6.%)	150 (11.2%)	89 (6.7%)	111 (8.3%)
	Healthy	Unhealthy	2 (0.1%)	33 (2.4%)	4 (0.3%)	8 (0.6%)
	Total worsened		195 (14.6%)	347 (25.9%)	184 (13.8%)	225 (16.8%)

## Supplementary Materials

The following are available online at https://www.mdpi.com/article/10.3390/ijerph191811772/s1, Supplementary Table S1. The analysis of covariance (ANCOVA) for the Adolescents’ Food Habits Checklist (AFHC) total score for the period before COVID-19 pandemic for place of residence, gender, age, body mass change and physical activity change in the total population of adolescents from the Diet and Activity of Youth during COVID-19 (DAY-19) Study (*n* = 1333), Supplementary Table S2. The analysis of covariance (ANCOVA) for the Adolescents’ Food Habits Checklist (AFHC) purchase score for the period before COVID-19 pandemic for place of residence, gender, age, body mass change and physical activity change in the total population of adolescents from the Diet and Activity of Youth during COVID-19 (DAY-19) Study (*n* = 1333), Supplementary Table S3. The analysis of covariance (ANCOVA) for the Adolescents’ Food Habits Checklist (AFHC) preparation score for the period before COVID-19 pandemic for place of residence, gender, age, body mass change and physical activity change in the total population of adolescents from the Diet and Activity of Youth during COVID-19 (DAY-19) Study (*n* = 1333), Supplementary Table S4. The analysis of covariance (ANCOVA) for the Adolescents’ Food Habits Checklist (AFHC) consumption score for the period before COVID-19 pandemic for place of residence, gender, age, body mass change and physical activity change in the total population of adolescents from the Diet and Activity of Youth during COVID-19 (DAY-19) Study (*n* = 1333), Supplementary Table S5. The analysis of covariance (ANCOVA) for the Adolescents’ Food Habits Checklist (AFHC) total score for the period of COVID-19 pandemic for place of residence, gender, age, body mass change and physical activity change in the total population of adolescents from the Diet and Activity of Youth during COVID-19 (DAY-19) Study (*n* = 1333), Supplementary Table S6. The analysis of covariance (ANCOVA) for the Adolescents’ Food Habits Checklist (AFHC) purchase score for the period of COVID-19 pandemic for place of residence, gender, age, body mass change and physical activity change in the total population of adolescents from the Diet and Activity of Youth during COVID-19 (DAY-19) Study (*n* = 1333), Supplementary Table S7. The analysis of covariance (ANCOVA) for the Adolescents’ Food Habits Checklist (AFHC) preparation score for the period of COVID-19 pandemic for place of residence, gender, age, body mass change and physical activity change in the total population of adolescents from the Diet and Activity of Youth during COVID-19 (DAY-19) Study (*n* = 1333), Supplementary Table S8. The analysis of covariance (ANCOVA) for the Adolescents’ Food Habits Checklist (AFHC) consumption score for the period of COVID-19 pandemic for place of residence, gender, age, body mass change and physical activity change in the total population of adolescents from the Diet and Activity of Youth during COVID-19 (DAY-19) Study (*n* = 1333).
