# Supplementary material for: Analysis of Association between Adolescents’ Food Habits and Body Mass Change in a Population-Based Sample: Diet and Activity of Youth during COVID-19 (DAY-19) Study
Source: Int J Environ Res Public Health. 2022 Sep 18;19(18):11772. doi: 10.3390/ijerph191811772 (PMC9517231; doi:10.3390/ijerph191811772)
Supplement: Supplementary file 1 [file ijerph-19-11772-s001.zip › ijerph-1850996-supplementary.pdf]

International Journal of
Environmental Research and Public Health

MDPI

Supplementary Materials

Analysis of Association between Adolescents' Food Habits and Body Mass Change in a Population-Based Sample: Diet and Activity of Youth during COVID-19 (DAY-19) Study

Aleksandra Kołota * and Dominika Głąbska

Supplementary Table S1. The analysis of covariance (ANCOVA) for the Adolescents' Food Habits Checklist (AFHC) total score for the period before COVID-19 pandemic for place of residence, gender, age, body mass change and physical activity change in the total population of adolescents from the Diet and Activity of Youth during COVID-19 (DAY-19) Study (n = 1333).

Source	Sum of squares	df	Mean square	*p*
Place of residence	0.00000	NaN	-	-
Gender	6.39e-14	0	-	-
Age	6.39e-14	0	-	-
Body mass change	0.00432	1	0.00432	0.769
Physical activity change	0.26461	1	0.26461	0.022
Place of residence ✻ gender	6.39e-14	0	-	-
Place of residence ✻ age	6.39e-14	0	-	-
Gender ✻ age	6.39e-14	0	-	-
Place of residence ✻ body mass change	1.07e0-4	1	1.07e-4	0.963
Gender ✻ body mass change	0.04221	1	0.04221	0.359
Age ✻ body mass change	0.01037	1	0.01037	0.649
Place of residence ✻ physical activity change	0.02230	1	0.02230	0.505
Gender ✻ physical activity change	0.00728	1	0.00728	0.703
Age ✻ physical activity change	0.02872	1	0.02872	0.449
Body mass change ✻ physical activity change	0.14733	3	0.04911	0.401
Place of residence ✻ gender ✻ age	6.39e-14	0	-	-
Place of residence ✻ gender ✻ body mass change	0.00289	1	0.00289	0.810
Place of residence ✻ age ✻ body mass change	0.00589	1	0.00589	0.732
Gender ✻ age ✻ body mass change	0.06697	1	0.06697	0.248
Place of residence ✻ gender ✻ physical activity change	0.03899	1	0.03899	0.378
Place of residence ✻ age ✻ physical activity change	0.07377	1	0.07377	0.225
Gender ✻ age ✻ physical activity change	0.06063	1	0.06063	0.272
Place of residence ✻ age ✻ physical activity change	0.11082	3	0.03694	0.530
Gender ✻ body mass change ✻ physical activity change	0.17067	3	0.05689	0.334
Age ✻ body mass change ✻ physical activity change	0.05114	3	0.01705	0.796
Place of residence ✻ gender ✻ age ✻ body mass change	0.04414	1	0.04414	0.348
Place of residence ✻ gender ✻ age ✻ physical activity change	0.02956	1	0.02956	0.443
Place of residence ✻ gender ✻ body mass change ✻ physical activity change	0.06934	3	0.02311	0.709
Place of residence ✻ age ✻ body mass change ✻ physical activity change	0.29832	3	0.09944	0.115
Gender ✻ age ✻ body mass change ✻ physical activity change	0.01840	3	0.00613	0.947
Place of residence ✻ gender ✻ age ✻ body mass change ✻ physical activity change	0.09059	3	0.03020	0.613
Residuals	63.25182	1262	0.05012	-

df – degrees of freedom; Nan – undefined operation.

Supplementary Table S2. The analysis of covariance (ANCOVA) for the Adolescents' Food Habits Checklist (AFHC) purchase score for the period before COVID-19 pandemic for place of residence, gender, age, body mass change and physical activity change in the total population of adolescents from the Diet and Activity of Youth during COVID-19 (DAY-19) Study (*n* = 1333).

Source	Sum of squares	df	Mean square	*p*
Place of residence	0.00000	NaN	-	-
Gender	-9.95e−14	0	-	-
Age	-9.95e−14	0	-	-
Body mass change	2.22e0-4	1	2.22e-4	0.949
Physical activity change	0.23065	1	0.23065	0.039
Place of residence ✻ gender	-9.95e−14	0	-	-
Place of residence ✻ age	-9.95e−14	0	-	-
Gender ✻ age	-9.95e−14	0	-	-
Place of residence ✻ body mass change	4.06e0-4	1	4.06e-4	0.931
Gender ✻ body mass change	0.04240	1	0.04240	0.375
Age ✻ body mass change	0.02937	1	0.02937	0.460
Place of residence ✻ physical activity change	0.10319	1	0.10319	0.167
Gender ✻ physical activity change	0.00881	1	0.00881	0.686
Age ✻ physical activity change	0.08946	1	0.08946	0.198
Body mass change ✻ physical activity change	0.10630	3	0.03543	0.578
Place of residence ✻ gender ✻ age	-9.95e−14	0	-	-
Place of residence ✻ gender ✻ body mass change	0.00899	1	0.00899	0.683
Place of residence ✻ age ✻ body mass change	1.09e0-4	1	1.09e-4	0.964
Gender ✻ age ✻ body mass change	0.15133	1	0.15133	0.094
Place of residence ✻ gender ✻ physical activity change	0.09803	1	0.09803	0.178
Place of residence ✻ age ✻ physical activity change	0.10027	1	0.10027	0.173
Gender ✻ age ✻ physical activity change	0.04585	1	0.04585	0.356
Place of residence ✻ age ✻ physical activity change	0.06654	3	0.02218	0.744
Gender ✻ body mass change ✻ physical activity change	0.09065	3	0.03022	0.641
Age ✻ body mass change ✻ physical activity change	0.03491	3	0.01164	0.885
Place of residence ✻ gender ✻ age ✻ body mass change	0.00532	1	0.00532	0.753
Place of residence ✻ gender ✻ age ✻ physical activity change	0.04139	1	0.04139	0.381
Place of residence ✻ gender ✻ body mass change ✻ physical activity change	0.02484	3	0.00828	0.927
Place of residence ✻ age ✻ body mass change ✻ physical activity change	0.58382	3	0.19461	0.013
Gender ✻ age ✻ body mass change ✻ physical activity change	0.12525	3	0.04175	0.508
Place of residence ✻ gender ✻ age ✻ body mass change ✻ physical activity change	0.07464	3	0.02488	0.709
Residuals	67.95772	1262	0.05385	-

df – degrees of freedom; Nan – undefined operation.

Supplementary Table S3. The analysis of covariance (ANCOVA) for the Adolescents' Food Habits Checklist (AFHC) preparation score for the period before COVID-19 pandemic for place of residence, gender, age, body mass change and physical activity change in the total population of adolescents from the Diet and Activity of Youth during COVID-19 (DAY-19) Study (n = 1333).

Source	Sum of squares	df	Mean square	p
Place of residence	0.00000	NaN	-	-
Gender	-2.84e−14	0	-	-
Age	-2.84e−14	0	-	-
Body mass change	0.03696	1	0.03696	0.459
Physical activity change	0.30478	1	0.30478	0.034
Place of residence ✻ gender	-2.84e−14	0	-	-
Place of residence ✻ age	-2.84e−14	0	-	-
Gender ✻ age	-2.84e−14	0	-	-
Place of residence ✻ body mass change	0.08387	1	0.08387	0.265
Gender ✻ body mass change	0.18365	1	0.18365	0.099
Age ✻ body mass change	0.06890	1	0.06890	0.312
Place of residence ✻ physical activity change	6.87e0-4	1	6.87e-4	0.920
Gender ✻ physical activity change	0.03768	1	0.03768	0.455
Age ✻ physical activity change	7.32e0-4	1	7.32e-4	0.917
Body mass change ✻ physical activity change	0.28236	3	0.09412	0.242
Place of residence ✻ gender ✻ age	-2.84e−14	0	-	-
Place of residence ✻ gender ✻ body mass change	0.00336	1	0.00336	0.823
Place of residence ✻ age ✻ body mass change	0.03589	1	0.03589	0.466
Gender ✻ age ✻ body mass change	5.98e0-4	1	5.98e-4	0.925
Place of residence ✻ gender ✻ physical activity change	0.01419	1	0.01419	0.646
Place of residence ✻ age ✻ physical activity change	0.05680	1	0.05680	0.359
Gender ✻ age ✻ physical activity change	0.08530	1	0.08530	0.261
Place of residence ✻ age ✻ physical activity change	0.37230	3	0.12410	0.138
Gender ✻ body mass change ✻ physical activity change	0.26578	3	0.08859	0.268
Age ✻ body mass change ✻ physical activity change	0.19780	3	0.06593	0.402
Place of residence ✻ gender ✻ age ✻ body mass change	0.21579	1	0.21579	0.074
Place of residence ✻ gender ✻ age ✻ physical activity change	0.06456	1	0.06456	0.328
Place of residence ✻ gender ✻ body mass change ✻ physical activity change	0.23311	3	0.07770	0.327
Place of residence ✻ age ✻ body mass change ✻ physical activity change	0.37943	3	0.12648	0.132
Gender ✻ age ✻ body mass change ✻ physical activity change	0.02022	3	0.00674	0.960
Place of residence ✻ gender ✻ age ✻ body mass change ✻ physical activity change	0.02982	3	0.00994	0.931
Residuals	85.07180	1262	0.06741	-

df – degrees of freedom; Nan – undefined operation.

Supplementary Table S4. The analysis of covariance (ANCOVA) for the Adolescents' Food Habits Checklist (AFHC) consumption score for the period before COVID-19 pandemic for place of residence, gender, age, body mass change and physical activity change in the total population of adolescents from the Diet and Activity of Youth during COVID-19 (DAY-19) Study (*n* = 1333).

Source	Sum of squares	df	Mean square	*p*
Place of residence	0.00000	NaN	-	-
Gender	0.00000	0	-	-
Age	0.00000	0	-	-
Body mass change	0.00169	1	0.00169	0.866
Physical activity change	0.08980	1	0.08980	0.218
Place of residence ✻ gender	0.00000	0	-	-
Place of residence ✻ age	0.00000	0	-	-
Gender ✻ age	0.00000	0	-	-
Place of residence ✻ body mass change	0.03509	1	0.03509	0.441
Gender ✻ body mass change	3.07e-4	1	3.07e-4	0.943
Age ✻ body mass change	0.03020	1	0.03020	0.474
Place of residence ✻ physical activity change	1.13e-7	1	1.13e-7	0.999
Gender ✻ physical activity change	0.00305	1	0.00305	0.820
Age ✻ physical activity change	0.04068	1	0.04068	0.407
Body mass change ✻ physical activity change	0.08753	3	0.02918	0.686
Place of residence ✻ gender ✻ age	0.00000	0	-	-
Place of residence ✻ gender ✻ body mass change	0.00309	1	0.00309	0.819
Place of residence ✻ age ✻ body mass change	0.02421	1	0.02421	0.522
Gender ✻ age ✻ body mass change	0.09130	1	0.09130	0.214
Place of residence ✻ gender ✻ physical activity change	0.00815	1	0.00815	0.710
Place of residence ✻ age ✻ physical activity change	0.00496	1	0.00496	0.772
Gender ✻ age ✻ physical activity change	0.01882	1	0.01882	0.572
Place of residence ✻ age ✻ physical activity change	0.21132	3	0.07044	0.311
Gender ✻ body mass change ✻ physical activity change	0.49399	3	0.16466	0.039
Age ✻ body mass change ✻ physical activity change	0.01990	3	0.00663	0.953
Place of residence ✻ gender ✻ age ✻ body mass change	0.00413	1	0.00413	0.791
Place of residence ✻ gender ✻ age ✻ physical activity change	0.00433	1	0.00433	0.787
Place of residence ✻ gender ✻ body mass change ✻ physical activity change	0.13257	3	0.04419	0.523
Place of residence ✻ age ✻ body mass change ✻ physical activity change	0.03303	3	0.01101	0.906
Gender ✻ age ✻ body mass change ✻ physical activity change	0.00301	3	0.00100	0.997
Place of residence ✻ gender ✻ age ✻ body mass change ✻ physical activity change	0.14735	3	0.04912	0.476
Residuals	74.46724	1262	0.05901	-

df – degrees of freedom; Nan – undefined operation.

Supplementary Table S5. The analysis of covariance (ANCOVA) for the Adolescents' Food Habits Checklist (AFHC) total score for the period of COVID-19 pandemic for place of residence, gender, age, body mass change and physical activity change in the total population of adolescents from the Diet and Activity of Youth during COVID-19 (DAY-19) Study (*n* = 1333).

Source	Sum of squares	df	Mean square	*p*
Place of residence	0.00000	NaN	-	-
Gender	2.84e-14	0	-	-
Age	2.84e-14	0	-	-
Body mass change	0.03517	1	0.03517	0.357
Physical activity change	0.15063	1	0.15063	0.057
Place of residence ✻ gender	2.84e-14	0	-	-
Place of residence ✻ age	2.84e-14	0	-	-
Gender ✻ age	2.84e-14	0	-	-
Place of residence ✻ body mass change	0.00780	1	0.00780	0.665
Gender ✻ body mass change	0.07831	1	0.07831	0.170
Age ✻ body mass change	0.00407	1	0.00407	0.754
Place of residence ✻ physical activity change	4.46e0-5	1	4.46e-5	0.974
Gender ✻ physical activity change	5.02e0-5	1	5.02e0-5	0.972
Age ✻ physical activity change	0.06960	1	0.06960	0.195
Body mass change ✻ physical activity change	0.16380	3	0.05460	0.267
Place of residence ✻ gender ✻ age	2.84e-14	0	-	-
Place of residence ✻ gender ✻ body mass change	0.02720	1	0.02720	0.418
Place of residence ✻ age ✻ body mass change	0.01947	1	0.01947	0.493
Gender ✻ age ✻ body mass change	0.05968	1	0.05968	0.230
Place of residence ✻ gender ✻ physical activity change	0.00257	1	0.00257	0.804
Place of residence ✻ age ✻ physical activity change	0.06868	1	0.06868	0.198
Gender ✻ age ✻ physical activity change	0.07593	1	0.07593	0.176
Place of residence ✻ age ✻ physical activity change	0.14728	3	0.04909	0.314
Gender ✻ body mass change ✻ physical activity change	0.19399	3	0.06466	0.197
Age ✻ body mass change ✻ physical activity change	0.06609	3	0.02203	0.661
Place of residence ✻ gender ✻ age ✻ body mass change	1.74e0-6	1	1.74e-6	0.995
Place of residence ✻ gender ✻ age ✻ physical activity change	9.50e0-4	1	9.50e-4	0.880
Place of residence ✻ gender ✻ body mass change ✻ physical activity change	0.18275	3	0.06092	0.221
Place of residence ✻ age ✻ body mass change ✻ physical activity change	0.06870	3	0.02290	0.647
Gender ✻ age ✻ body mass change ✻ physical activity change	0.05639	3	0.01880	0.715
Place of residence ✻ gender ✻ age ✻ body mass change ✻ physical activity change	0.19927	3	0.06642	0.187
Residuals	52.31015	1262	0.04145	-

df – degrees of freedom; Nan – undefined operation.

Supplementary Table S6. The analysis of covariance (ANCOVA) for the Adolescents' Food Habits Checklist (AFHC) purchase score for the period of COVID-19 pandemic for place of residence, gender, age, body mass change and physical activity change in the total population of adolescents from the Diet and Activity of Youth during COVID-19 (DAY-19) Study (*n* = 1333).

Source	Sum of squares	df	Mean square	*p*
Place of residence	0.00000	NaN	-	-
Gender	-4.97e−14	0	-	-
Age	-4.97e−14	0	-	-
Body mass change	0.03564	1	0.03564	0.398
Physical activity change	0.11307	1	0.11307	0.132
Place of residence ✻ gender	-4.97e−14	0	-	-
Place of residence ✻ age	-4.97e−14	0	-	-
Gender ✻ age	-4.97e−14	0	-	-
Place of residence ✻ body mass change	0.00244	1	0.00244	0.825
Gender ✻ body mass change	0.22723	1	0.22723	0.033
Age ✻ body mass change	0.00722	1	0.00722	0.704
Place of residence ✻ physical activity change	0.01869	1	0.01869	0.541
Gender ✻ physical activity change	0.02468	1	0.02468	0.482
Age ✻ physical activity change	0.06074	1	0.06074	0.270
Body mass change ✻ physical activity change	0.30800	3	0.10267	0.104
Place of residence ✻ gender ✻ age	-4.97e−14	0	-	-
Place of residence ✻ gender ✻ body mass change	0.02523	1	0.02523	0.477
Place of residence ✻ age ✻ body mass change	0.00800	1	0.00800	0.689
Gender ✻ age ✻ body mass change	0.10441	1	0.10441	0.148
Place of residence ✻ gender ✻ physical activity change	0.00374	1	0.00374	0.784
Place of residence ✻ age ✻ physical activity change	0.13923	1	0.13923	0.095
Gender ✻ age ✻ physical activity change	0.02309	1	0.02309	0.496
Place of residence ✻ age ✻ physical activity change	0.19917	3	0.06639	0.263
Gender ✻ body mass change ✻ physical activity change	0.22278	3	0.07426	0.216
Age ✻ body mass change ✻ physical activity change	0.04271	3	0.01424	0.836
Place of residence ✻ gender ✻ age ✻ body mass change	0.00589	1	0.00589	0.731
Place of residence ✻ gender ✻ age ✻ physical activity change	0.00662	1	0.00662	0.716
Place of residence ✻ gender ✻ body mass change ✻ physical activity change	0.43397	3	0.14466	0.034
Place of residence ✻ age ✻ body mass change ✻ physical activity change	0.17609	3	0.05870	0.317
Gender ✻ age ✻ body mass change ✻ physical activity change	0.16669	3	0.05556	0.342
Place of residence ✻ gender ✻ age ✻ body mass change ✻ physical activity change	0.21494	3	0.07165	0.230
Residuals	62.92438	1262	0.04986	-

df – degrees of freedom; Nan – undefined operation.

Supplementary Table S7. The analysis of covariance (ANCOVA) for the Adolescents' Food Habits Checklist (AFHC) preparation score for the period of COVID-19 pandemic for place of residence, gender, age, body mass change and physical activity change in the total population of adolescents from the Diet and Activity of Youth during COVID-19 (DAY-19) Study (n = 1333).

Source	Sum of squares	df	Mean square	p
Place of residence	0.00000	NaN	-	-
Gender	-5.68e−14	0	-	-
Age	-5.68e−14	0	-	-
Body mass change	0.01133	1	0.01133	0.643
Physical activity change	0.11021	1	0.11021	0.149
Place of residence ✻ gender	-5.68e−14	0	-	-
Place of residence ✻ age	-5.68e−14	0	-	-
Gender ✻ age	-5.68e−14	0	-	-
Place of residence ✻ body mass change	0.02124	1	0.02124	0.526
Gender ✻ body mass change	0.05327	1	0.05327	0.315
Age ✻ body mass change	0.00302	1	0.00302	0.811
Place of residence ✻ physical activity change	0.00657	1	0.00657	0.724
Gender ✻ physical activity change	0.00325	1	0.00325	0.804
Age ✻ physical activity change	0.03179	1	0.03179	0.438
Body mass change ✻ physical activity change	0.26789	3	0.08930	0.167
Place of residence ✻ gender ✻ age	-5.68e−14	0	-	-
Place of residence ✻ gender ✻ body mass change	0.02318	1	0.02318	0.508
Place of residence ✻ age ✻ body mass change	0.06988	1	0.06988	0.250
Gender ✻ age ✻ body mass change	0.02394	1	0.02394	0.501
Place of residence ✻ gender ✻ physical activity change	0.00201	1	0.00201	0.845
Place of residence ✻ age ✻ physical activity change	0.05821	1	0.05821	0.294
Gender ✻ age ✻ physical activity change	0.08897	1	0.08897	0.194
Place of residence ✻ age ✻ physical activity change	0.14559	3	0.04853	0.431
Gender ✻ body mass change ✻ physical activity change	0.16859	3	0.05620	0.363
Age ✻ body mass change ✻ physical activity change	0.13465	3	0.04488	0.466
Place of residence ✻ gender ✻ age ✻ body mass change	4.73e0-6	1	4.73e0-6	0.992
Place of residence ✻ gender ✻ age ✻ physical activity change	9.50e0-4	1	9.50e0-4	0.893
Place of residence ✻ gender ✻ body mass change ✻ physical activity change	0.14731	3	0.04910	0.425
Place of residence ✻ age ✻ body mass change ✻ physical activity change	0.03856	3	0.01285	0.866
Gender ✻ age ✻ body mass change ✻ physical activity change	0.10018	3	0.03339	0.594
Place of residence ✻ gender ✻ age ✻ body mass change ✻ physical activity change	0.34342	3	0.07165	0.090
Residuals	66.61883	1262	0.11447	-

df – degrees of freedom; Nan – undefined operation.

Supplementary Table S8. The analysis of covariance (ANCOVA) for the Adolescents' Food Habits Checklist (AFHC) consumption score for the period of COVID-19 pandemic for place of residence, gender, age, body mass change and physical activity change in the total population of adolescents from the Diet and Activity of Youth during COVID-19 (DAY-19) Study (*n* = 1333).

Source	Sum of squares	df	Mean square	*p*
Place of residence	0.00000	NaN	-	-
Gender	0.00000	0	-	-
Age	0.00000	0	-	-
Body mass change	0.03749	1	0.03749	0.420
Physical activity change	0.16930	1	0.16930	0.086
Place of residence ✻ gender	0.00000	0	-	-
Place of residence ✻ age	0.00000	0	-	-
Gender ✻ age	0.00000	0	-	-
Place of residence ✻ body mass change	9.80e-4	1	9.80e-4	0.896
Gender ✻ body mass change	0.05922	1	0.05922	0.310
Age ✻ body mass change	0.01465	1	0.01465	0.614
Place of residence ✻ physical activity change	0.00941	1	0.00941	0.686
Gender ✻ physical activity change	0.00338	1	0.00338	0.809
Age ✻ physical activity change	0.08640	1	0.08640	0.221
Body mass change ✻ physical activity change	0.07536	3	0.02512	0.727
Place of residence ✻ gender ✻ age	0.00000	0	-	-
Place of residence ✻ gender ✻ body mass change	0.00582	1	0.00582	0.750
Place of residence ✻ age ✻ body mass change	0.02443	1	0.02443	0.515
Gender ✻ age ✻ body mass change	0.09306	1	0.09306	0.204
Place of residence ✻ gender ✻ physical activity change	3.15e-6	1	3.15e-6	0.994
Place of residence ✻ age ✻ physical activity change	0.00248	1	0.00248	0.836
Gender ✻ age ✻ physical activity change	0.02924	1	0.02924	0.476
Place of residence ✻ age ✻ physical activity change	0.18963	3	0.06321	0.348
Gender ✻ body mass change ✻ physical activity change	0.37792	3	0.12597	0.087
Age ✻ body mass change ✻ physical activity change	0.00651	3	0.00217	0.990
Place of residence ✻ gender ✻ age ✻ body mass change	0.00299	1	0.00299	0.820
Place of residence ✻ gender ✻ age ✻ physical activity change	1.26e-6	1	1.26e-6	0.996
Place of residence ✻ gender ✻ body mass change ✻ physical activity change	0.19399	3	0.06466	0.338
Place of residence ✻ age ✻ body mass change ✻ physical activity change	0.02373	3	0.00791	0.938
Gender ✻ age ✻ body mass change ✻ physical activity change	0.06430	3	0.02143	0.773
Place of residence ✻ gender ✻ age ✻ body mass change ✻ physical activity change	0.23582	3	0.07861	0.251
Residuals	72.56583	1262	0.05750	-

df – degrees of freedom; Nan – undefined operation.
